# BigSolDB 2.0, dataset of solubility values for organic compounds in different solvents at various temperatures

**DOI:** 10.1038/s41597-025-05559-8

**Published:** 2025-07-15

**Authors:** Lev Krasnov, Dmitry Malikov, Marina Kiseleva, Sergei Tatarin, Sergey Sosnin, Stanislav Bezzubov

**Affiliations:** 1 N.S. Kurnakov Institute of General and Inorganic Chemistry, Russian Academy of Sciences, Leninskii pr. 31, 119991 Moscow, Russia; 2 Department of Chemistry, M.V. Lomonosov Moscow State University, Lenin’s Hills 1/3, 119991 Moscow, Russia; 3 Department of Pharmaceutical Sciences, University of Vienna, Josef-Holaubek-Platz 2, 1090 Vienna, Austria; 4 National Research University Higher School of Economics, 7 Vavilova Str., Moscow, 117312 Russia

**Keywords:** Cheminformatics, Physical chemistry

## Abstract

Solubility is one of the key properties of organic compounds that determines their applications in chemistry, materials science and pharmaceuticals. However, predicting solubility values in any solvent except water from a molecular structure still remains a challenging task in modern cheminformatics, not least due to the lack of large and diverse datasets. In this study, we present a dataset containing 103944 experimental solubility values within a temperature range from 243 to 425 K for 1448 organic compounds measured in 213 individual solvents extracted from 1595 peer-reviewed articles. The molecular structures of solutes and solvents as well as solubility data are standardized and provided in a machine-readable format, allowing straightforward data-driven analysis. We have also developed a web-tool for interactive visualization and search within the dataset. This dataset can serve as a comprehensive benchmark for developing machine learning for predicting solubility.

## Background & Summary

Solubility is an extremely important parameter, which determines the applicability of a certain compound in synthetic, medicinal, environmental chemistry, geochemistry and many other chemistry-related research and industry. In particular, organic solvents for synthesis are often chosen considering the solubility of initial reagents to boost the reaction itself or ensure low solubility of the desired product facilitating its isolation^1–3^. Moreover, the aqueous solubility is crucial for design of pharmaceutics, as it significantly limits bioavailability of drugs^4^. Solubility is also critical for the development of effective extraction and crystallization methods^5,6^. Given the need to perform screening of large libraries of compounds, experimentally measuring the solubility of each compound seems to be an incredibly tedious process sometimes even proves infeasible. Thus, there is a clear need for a tool for the direct assessment of molecular solubility from chemical structures of a solute-solvent pair.

The prediction of solubility still represents a significant challenge in modern quantum chemistry and cheminformatics, and dozens of various methods have been developed to fill this gap. The computational approaches usually include the use of molecular dynamics^7,8^ and/or ab initio calculations^9,10^. Various data-driven approaches have also emerged, seeking to establish Quantitative Structure-Activity Relationship (QSAR)^11^ through the development of machine learning (ML) models. The importance of this task has also been highlighted by two solubility challenges to the cheminformatics community^12,13^. It is worth mentioning that most studies considered only aqueous solubility^14–18^. Accordingly, the data surveys were also predominantly focused on the solubility in water, with AqSolDB being the most notable contribution to the field^19,20^. Later, attempts to predict solubility in organic solvents were made possible by rational selection of descriptors and curation/usage of corresponding datasets^21–25^, which particularly emerged with the release of the previous version of BigSolDB^26–32^.

There is a strong claim that availability of good datasets, both in terms of quality and quantity of entries, is one of the key features toward successful predictions^19,28,33^, although it is not the exclusive one^34,35^. In particular, it was shown that post-processing of AqSolDB resulted in improvement of prediction metrics, and the importance of high-quality data was also underlined^36^. Moreover, the researchers highlighted scarcity of comprehensive datasets for organic solubility^32^. Thus, in our work we aimed at creating the largest up-to-date dataset of solubility values of organic compounds, expanding BigSolDB to version 2.0, reaching 103944 values for 1448 compounds in 213 solvents from 1595 peer-reviewed articles. We believe that this dataset will provide a solid starting point for training machine learning and deep learning models for predicting solubility.

## Methods

**Fig. 1:**
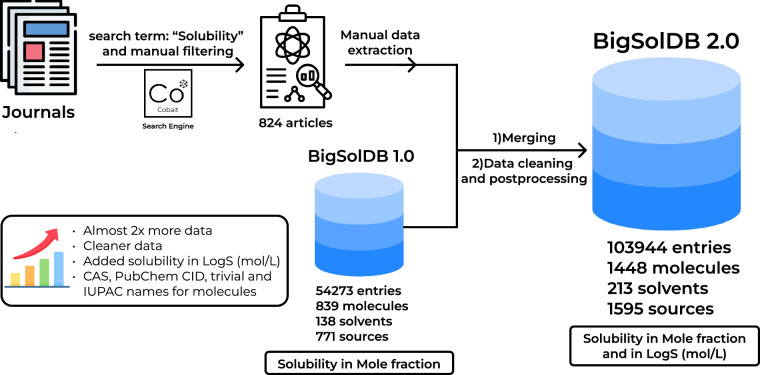
The general scheme of BigSolDB 2.0 construction.

In this research, BigSolDB 1.0^26^ was significantly expanded, as shown in the general scheme (Fig. 1). First, from the peer-reviewed journals, which usually publish articles considering physico-chemical properties of molecules and solutions (which included Journal of Molecular Liquids, Journal of Solution Chemistry, and underexplored in version 1.0. domains of Journal of Chemical & Engineering Data, Chinese Journal of Chemical Engineering and Fluid Phase Equilibria), articles containing the word “solubility” in the title were extracted using freely available Cobalt search engine (an example of search query is as follows: https://cobalt.colab.ws/?term=solubility&publisher_id=17&journal_id=13597). The extracted articles were manually screened for solubility data. Only articles which directly report molar solubility expressed in mole fraction were picked to avoid additional mistakes upon conversion. Most of the solubility values in this dataset were measured using common thermodynamic methods^37^, from which various modifications of Saturation Shake-Flask, Dissolution or Potentiometric methods can be highlighted. All molecular structures were converted to SMILES format using PubChem (https://pubchem.ncbi.nlm.nih.gov/). If the solute molecule was not presented in the PubChem database, the corresponding SMILES was generated by ChemDraw 18.0. The SMILES were canonized using RDKit (https://www.rdkit.org). After that the dataset was united with BigSolDB 1.0. and subjected to cleaning and other post-processing.

The processing of data included:

- Unification of solvent designations in order to eliminate ambiguity. As an illustrative example, “2-butanol”, “butan-2-ol”, and “sec-butyl alcohol” were converted to “sec-butanol”. After that, the SMILES of solvents were added using PubChem. Note that in some entries polymeric solvents were used (i.e. PEG-400), for these SMILES were not added.
- Cleaning duplicates. During the dataset construction it was found that there are some entries which contain exactly the same data (solute, solvent, temperature, solubility value) but with different data sources and no provided links between each other. For these, the value reported in the older article was usually left while the data in the newer article was deleted. It should be noted that such procedure was performed only for the articles which display perfectly identical values (which probably represent the same measurement), whereas, following recommendations from the literature^34^, individual solubility measurements from different publications are preserved in BigSolDB.
- Conversion to LogS units. In order to make the distribution of data more uniform as usually desirable for ML applications, another unit system is needed. The most commonly used data representation is LogS, where S stands for molar solubility (mol/L). Firstly, molecular weight of solvents was automatically counted with RDkit. Another task to perform the conversion was obtaining solution density values at various temperatures. Since it was claimed that an approximate conversion, using the density of the solvent at the specified temperature as a stand-in for the density of the solution, is valid for data with LogS  < 0.5^30^, which constitutes the majority of the dataset, we sought densities of pure solvents at various temperatures instead. We extracted the experimental values of densities of solvents, for which there are  > 100 entries in the BigSolDB 2.0, from peer-reviewed scientific articles. For the solubility data points for which we had the exact experimental values of solvent densities, these were used to calculate LogS. To evaluate the solvent density at any other temperature (for which the exact experimental value was not found) we built linear approximations using the abovementioned experimental points. The experimental values for every graph were brought from  > = 2 articles and R^2^ for each such approximation was  > 0.995.
- Adding extra information about the solutes. Namely, CAS numbers, names as well as PubChem CID were extracted using PubChem API (https://github.com/mcs07/PubChemPy). The information if the solute is an FDA-approved drug was extracted using ChEMBL webresource client (https://github.com/chembl/chembl_webresource_client). Note that in some cases we opted for trivial names instead of IUPAC names, if such existed.

## Data Records

BigSolDB 2.0 can be accessed online within Zenodo^38^. The main dataset is structured as a downloadable CSV format data record. Description of each available metadata field is provided in Table 1. The additional dataset of solvents densities values is also provided as a downloadable CSV format data record, its description is provided in Table 2.

**Table 1:** Description of each metadata field for the main dataset.

Column	Description	Type
SMILES_Solute	SMILES representation of the solute molecule	string
Temperature_K	temperature for the reported solubility value, K	float
Solvent	solvent name	string
SMILES_Solvent	SMILES representation of the solvent molecule	string
Solubility(mole_fraction)	the reported solubility value expressed in mole fraction of solute	float
Solubility(mol/L)	the recalculated solubility value expressed in molar concentration of solute (mol/L)	float
LogS(mol/L)	decimal logarithm of the recalculated solubility value expressed in molar concentration of solute (mol/L)	float
Compound_Name	solute name	string
CAS	solute CAS number	string
PubChem_CID	solute PubChem CID	string
FDA_Approved	designation if the solute is a FDA-approved drug. ‘Yes’ is stated for FDA approved drugs while ‘No’ is stated for others	string
Source	DOI of a data source for given values	string

**Table 2:** Description of each metadata field for the solvents densities dataset.

Solvent	solvent name	string
Temperature_K	temperature for the reported density value, K	float
Density_g/cm ∧ 3	the reported density value	float
Source	data source for given values	string

**Fig. 2:**
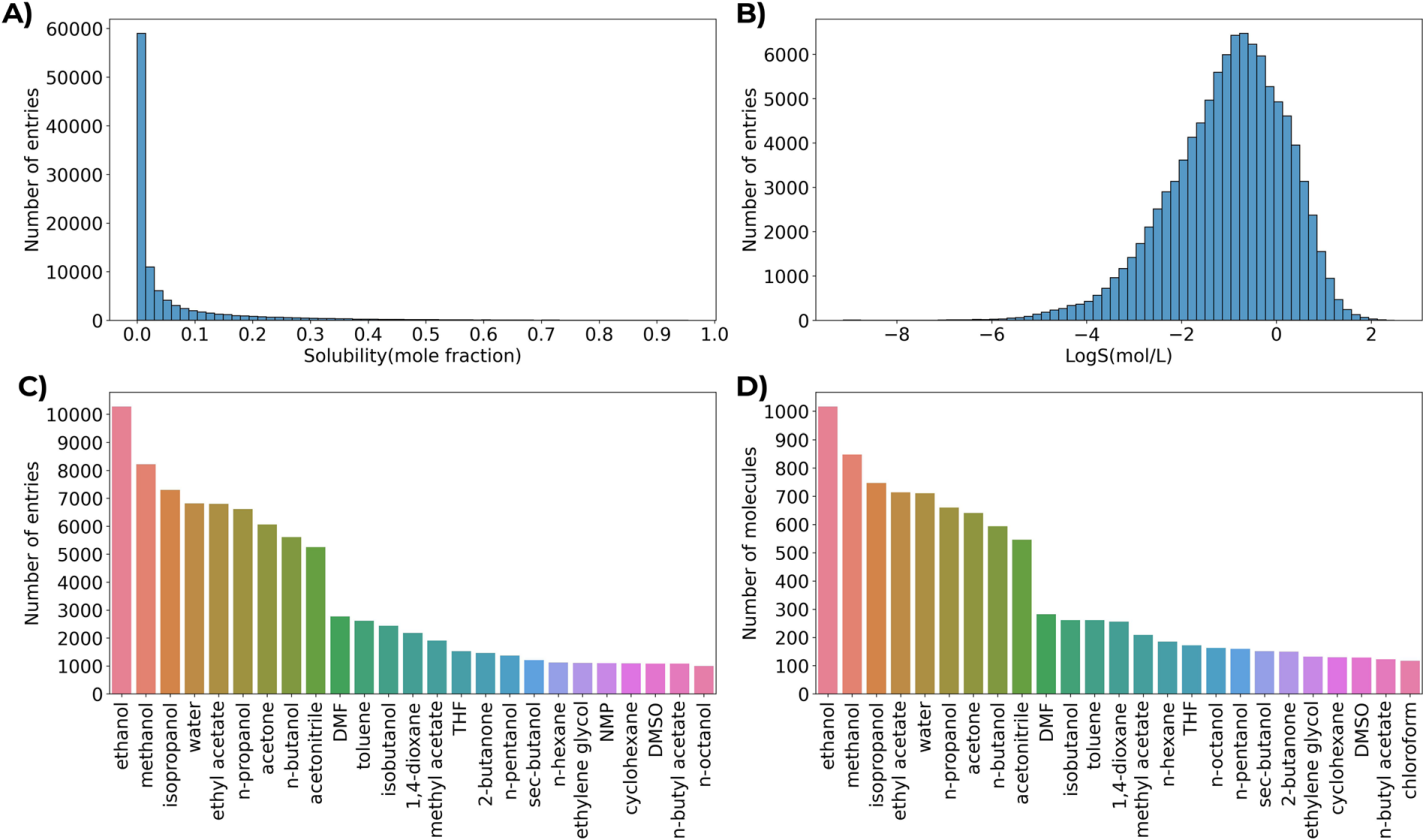
Statistics in the BigSolDB 2.0. **A**) Distribution of entries based on mole fraction solubility values. **B**) Distribution of entries based on LogS(mol/L) solubility values. **C**) Distribution of the solvent media by number of entries (top 25 solvents are presented). **D**) Distribution of the solvent media by number of unique solutes (top 25 solvents are presented).

The detailed statistics of the dataset are presented in Fig. 2. One can notice that the vast majority of molecules display relatively low absolute solubility values with mole fraction  < 0.01 (52677 entries), so such distribution is not really representative (Fig. 2A). Indeed, logarithmic representation is far more uniform, with a distinct maximum at LogS(mol/L) = −1 and ends tailing to LogS(mol/L) = −9 in the region of extremely low solubility and to LogS(mol/L) = 2 in the region of extremely high solubility, respectively (Fig. 2B). From the most common solvents, both in terms of unique entries and unique solute molecules, low-molecular weight alcohols, water, ethyl acetate, acetone and acetonitrile clearly stand out (Fig. 2C,D). Considering the noise level estimation, the dataset contains 6939 entries corresponding to 176 individual solutes for which multiple independent measurements in the same conditions (solvent, temperature) are presented. The pairwise comparison of these measurements yields an RMSE of 0.39 logS units and an R^2^ of 0.90. This indicates a relatively high internal consistency of literature data in BigSolDB, and suggests slightly lower variability compared to the noise levels previously estimated for literature solubility measurements by Mitchell et al^34^.

## Technical Validation

As the presented experimental data has been already published in peer-reviewed scientific journals, the only potential errors in our dataset can be formally related to mistakes in data extraction and summarization. To potentially avoid such errors and ensure consistency and validity of the dataset, we have performed cross-checking of the data, adapted from^39^. Two people with sufficient experience in physical chemistry separately collected the solubility data from the papers. The third person checked these two datasets and added them to the final dataset. The inconsistencies were double-checked, and the correct variant was added. After that the resulting dataset was subjected to a rechecking to ensure completeness and clarity of the data. The process of rechecking the dataset was as follows:

- We have checked and fixed all entries with error values of mole fraction values exceeding 1, which are physically impossible.
- We have cleaned values with abnormal temperature values (i.e. with temperature  > boiling temperature of the solvents, which is the case for studies of solubility in subcritical fluids).
- We have checked the validity of all sources by checking all DOIs using CrossRef API (https://github.com/CrossRef/rest-api-doc).
- We have additionally checked the cases when two or more sets of values were measured for the same solute-solvent pair. In such cases we have reassessed the articles in which huge (>=1 magnitude mole fraction) differences in the obtained values were observed in order to make sure that the data extraction process was correct.
- We have checked cases in which for 1 unique name 2 different SMILES were observed and vice versa. This was done to eliminate potentially incorrect ‘Compound_Name’ and ‘SMILES_Solute’ designations.

## Usage Notes

The main objective of collecting and sharing these data is to propose a benchmark dataset for 1) straightforward evaluation, visualization and analysis of solubility data for various organic molecules 2) accelerating research in the field of data-driven solubility estimation. Moreover, this dataset paves the way to train machine learning models for solubility prediction in a wide variety of organic solvents which can be extremely useful in the design of many chemical and technological processes.

In order to present fast and convenient method of navigation through the dataset, we have also developed an online tool (https://bigsoldb.streamlit.app/). It allows one to easily search (both by chemical structure and by trivial name) and render solubility values for organic compounds.

Further development of the dataset may constitute from literature screenings as well as from standardized data contributions from the laboratories from all around the world. Both these types of data are highly desired in the future to provide even more large and diverse dataset for solubility prediction purposes.

## Data Availability

The code used in this study is available at https://github.com/levakrasnovs/BigSolDBv2.0/tree/main.
